# Inpatient and Outpatient Radiology Report Access After the 21st Century Cures Act

**DOI:** 10.1001/jamanetworkopen.2025.28683

**Published:** 2025-08-27

**Authors:** Jordan R. Pollock, Amara Tariq, John J. Schmitz, Jacob Varner, Umesh Sharma, Allie M. Metcalfe, Nelly Tan

**Affiliations:** 1 Department of Radiology, Mayo Clinic, Phoenix, Arizona; 2 Department of Radiology, Mayo Clinic, Rochester, Minnesota; 3 Department of Internal Medicine, Mayo Clinic, Phoenix, Arizona; 4 Department of Radiology, Mayo Clinic, Jacksonville, Florida

## Abstract

This cohort study examines trends in patient access of radiology imaging results after implementation of the 21st Century Cures Act in different clinical settings.

## Introduction

The information-blocking provisions of the 21st Century Cures Act (hereafter, Cures Act) Final Rule mandate that all health care organizations immediately release test results to patients.^1^ Before the Cures Act, test results were delivered to patients following an institutional-dependent delay to allow for care coordination and delivery of these results.^2^ The purpose of this study was to examine trends in patient access of radiology imaging results after implementation of the Cures Act between different clinical settings.

## Methods

This cohort study was approved by the Mayo Clinic institutional review board and is a multicenter, retrospective, before-and-after, Health Insurance Portability and Accountability Act (HIPAA)–compliant study conducted from January 2021 to December 2022. Given that the data were deidentified and aggregated, informed consent was not required, in accordance with 45 CFR §46. Beginning January 15, 2022, any finalized radiology reports were sent immediately to the ordering practitioner and the patient, rather than having a 36-hour delay for patients. Our study follows the STROBE reporting guidelines, and data were analyzed using Stata version 19.0 (StataCorp LLC). For patient selection process flowchart and patient and/or examination characteristics, please see eFigure and eTable in Supplement 1. Race was self-reported in the electronic health record and is included in this study to provide further information about the patient population studied and generalizability of our results to other institutions. We compared the median access time of finalized radiology reports for inpatient and/or emergency department patients vs outpatients before and after implementation of the Cures Act. We aimed to specifically investigate trends among patients who access radiology reports promptly. For this purpose, we selected reports that were accessed within the first 24 hours of release. Percentage change was calculated before and after implementation of the Cures Act.

## Results

In total, there were 84 144 patients and 242 902 examinations for inpatient and emergency department analysis. In the outpatient group, there were 335 869 patients and 1 005 837 examinations. The Table and the Figure show the median hours it took patients before vs after Cures Act implementation in the inpatient and/or emergency department setting compared with outpatient setting to access their reports after report release. The median (IQR) time to access report after release for those who accessed their reports within 24 hours among the inpatient and/or emergency department population decreased from 9.1 (4.2-14.1) hours to 2.8 (0.7-9.2) hours (−69.2%). The outpatient population experienced a median (IQR) decrease from 4.9 (1.1-8.9) hours to 1.1 (0.2-4.7) hours (−77.6%). These substantial decreases do not include the removal of the 36-hour embargo after implementation of the Cures Act (Table and Figure).

**Table.** Time for Patients to Access Their Reports Before vs After 21st Century Cures Act Implementation^a^

	Inpatient and/or emergency department				Outpatient			
	Before Cures Act (IQR)	Before Cures Act with 36-h embargo (IQR)	After Cures Act (IQR)	Change (with 36-h embargo)	Before Cures Act (IQR)	Before Cures Act with 36-h embargo (IQR)	After Cures Act (IQR)	Change (with 36-h embargo)
Overall	9.1 (4.2-14.1)	45.1 (40.2-50.1)	2.8 (0.7-9.2)	_−_69 (_−_94)	4.9 (1.1-8.9)	40.9 (37.1-44.9)	1.1 (0.2-4.7)	_−_78 (_−_97)
Race								
Asian	9.3 (4.1_⁻_14.0)	45.3 (40.1-50.0)	2.2 (0.6-6.9)	_−_76 (_−_95)	4.1 (0.6-8.5)	40.1 (36.6-44.5)	1.0 (0.1-4.2)	_−_76 (_−_98)
Black	9.3 (4.3-14.4)	45.3 (40.3-50.4)	3.7 (0.9-11.3)	_−_60 (_−_92)	4.1 (0.7-8.7)	40.1 (36.7-44.7)	1.4 (0.2-5.6)	_−_66 (_−_97)
White	9.0 (4.2-14.0)	45.0 (40.2-50.0)	2.8 (0.7-9.2)	_−_69 (_−_94)	4.9 (1.1-8.9)	40.9 (37.1-44.9)	1.1 (0.2-4.7)	_−_78 (_−_97)
Other	9.6 (4.6-14.4)	45.6 (40.6-50.6)	3.4 (0.9-10.1)	_−_65 (_−_93)	4.4 (0.7-8.5)	40.4 (36.7-44.5)	1.2 (0.2-4.9)	_−_73 (_−_97)
Age group, y								
<30	9.1 (4.0-14.6)	45.1 (40.0-50.6)	2.3 (0.5-7.8)	_−_75 (_−_95)	2.8 (0.2-7.1)	38.8 (36.2-43.1)	0.4 (0.03- 2.4)	_−_88 (_−_99)
30-50	8.8 (3.9-13.9)	44.8 (39.9-49.9)	2.4 (0.6-8.3)	_−_73 (_−_95)	3.5 (0.5-7.5)	39.5 (36.5-43.5)	0.6 (0.1-2.9)	_−_84 (_−_99)
50-70	9.1 (4.2-14.1)	45.1 (40.2-50.1)	2.9 (0.8-9.6)	_−_68 (_−_94)	5.1 (1.2-9.0)	41.1 (37.2-45.0)	1.2 (0.2-4.9)	_−_77 (_−_97)
>70	6.3 (4.5-14.1)	42.3 (40.5-50.1)	3.2 (0.9-10.4)	_−_49 (_−_92)	6.3 (2.4-10.0)	42.3 (38.4-46.0)	2.1 (0.5-6.8)	_−_67 (_−_95)
Gender								
Male	9.0 (4.1-14.0)	45.0 (40.1-50.0)	2.9 (0.8-9.0)	_−_68 (_−_94)	5.1 (1.3-9.0)	41.1 (37.3-45.0)	1.2 (0.2-5.0)	_−_77 (97)
Female	9.1 (4.3-14.2)	45.1 (40.3-50.2)	2.8 (0.7-9.4)	_−_69 (_−_94)	4.7 (0.9-8.7)	40.7 (36.9-44.7)	1.1 (0.2-4.4)	_−_77 (97)
Insurance type								
Commerical	8.9 (4.0-14.0)	44.9 (40.0-50.0)	2.6 (0.6-8.7)	_−_71 (_−_94)	4.2 (0.7-8.2)	40.2 (36.7-44.2)	0.8 (0.1-3.9)	_−_81 (98)
Medicare or Medicaid	9.2 (4.5-14.2]	45.2 (40.5-50.2]	3.1 (2.0-9.6]	_−_66 (_−_93)	5.8 (1.9-9.6]	41.8 (37.9-45.6]	1.8 (0.4-6.2]	_−_69 (96)

^a^ Table includes only patients who accessed their reports in the first 24 hours of release.

^b^ Other race includes American Indian and/or Alaska Native, Hispanic, Native Hawaiian and/or other Pacific Islander, and two or more races.

**Figure.**
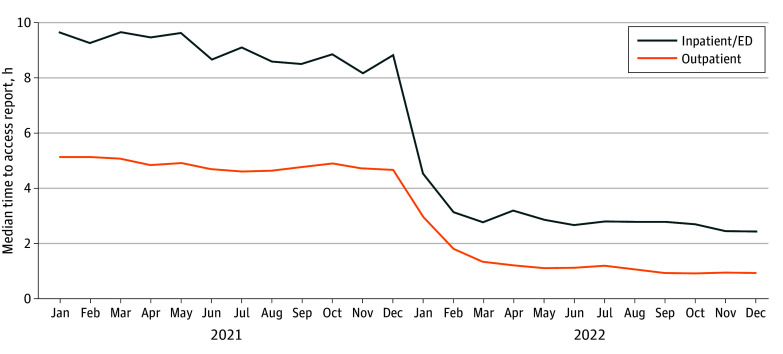
Difference in Median Time to Access Report Before vs After 21st Century Cures Act Implementation in January 2022 This figure shows the difference in median time to access report (in hours) before vs after 21st Century Cures Act implementation in January 2022 at our institution among all patients who accessed their reports within 24 hours of report release. This figure does not include the 36-hour embargo that existed before Cures Act implementation. ED indicates emergency department.

## Discussion

The Cures Act has been met with mixed reviews by patients and practitioners. A recent survey of 8139 patients in the US found that 95.7% of respondents would prefer to receive results as soon as they were available, even if the ordering practitioner has not reviewed the result.^3^ Interviews and surveys of 29 clinicians and 29 patients involved with cancer care found that patients generally felt more positive about the Cures Act information-blocking rule than clinicians, reporting benefits such as relief, gratitude, and empowerment.^4^ Notwithstanding, this policy has a few unintended consequences, such as patients reviewing their test results before their practitioner, patients discovering sensitive health results (ie, cancer) electronically rather than in person, and pressure for practitioners to contact patients quickly. For example, a study of 249 ordering practitioners found that 83.8% of ordering practitioners reported an increase in patient call volume after removing the embargo to comply with the information-blocking Cures Act.^5^ A limitation of this cohort study is that the data are from a single institution and trends observed may not apply to other institutions.

After implementation of the Cures Act, patients in the outpatient setting viewed their examination results substantially quicker than patients in the inpatient and/or emergency department settings. Ordering practitioners and radiologists should be aware that patients receive immediate access to finalized imaging reports, often within a few hours of result release.

## Notes

Supplement 1. **eFigure.** STROBE Diagram **eTable.** This table demonstrates the demographics of our study population including race, age, gender, insurance coverage, and imaging modality

Supplement 2.
Data Sharing Statement
