# Supplementary material for: Inpatient and Outpatient Radiology Report Access After the 21st Century Cures Act
Source: JAMA Netw Open. 2025 Aug 27;8(8):e2528683. doi: 10.1001/jamanetworkopen.2025.28683 (PMC12391978; doi:10.1001/jamanetworkopen.2025.28683)
Supplement: Supplement 1. — eFigure. STROBE Diagram eTable. This table demonstrates the demographics of our study population including race, age, gender, insurance coverage, and imaging modality [file jamanetwopen-e2528683-s001.pdf]

Supplemental Online Content

Pollock JR, Tariq A, Schmitz JJ, et al. Inpatient and outpatient radiology report access after the 21st Century Cures Act. *JAMA Netw Open.* 2025;8(8):e2528683. doi:10.1001/jamanetworkopen.2025.28683

eFigure. STROBE Diagram

eTable. This table demonstrates the demographics of our study population including race, age, gender, insurance coverage, and imaging modality

This supplemental material has been provided by the authors to give readers additional information about their work.

© 2025 Pollock JR et al. *JAMA Network Open*.

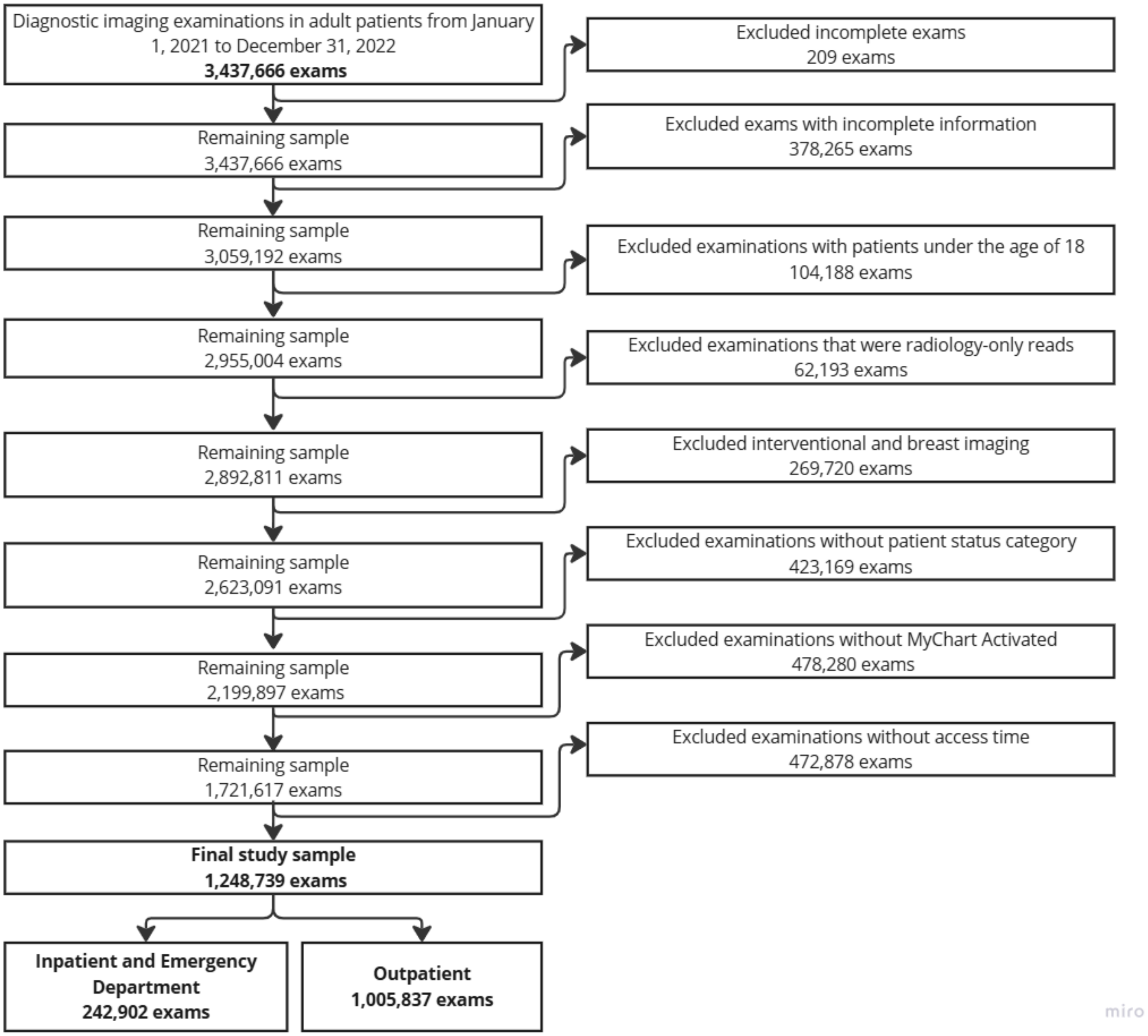

eFigure. STROBE Diagram

© 2025 Pollock JR et al. *JAMA Network Open*.

	INPATIENT/ED		OUTPATIENT	
	**Reports**	**Patients**	**Reports**	**Patients**
**TOTAL**	**242902**	**84144**	**1005837**	**335869**
**RACE**				
White	217217 (89.4%)	75289 (89.4%)	917637 (91.2%)	304076 (90.5%)
Black	10759 (4.4%)	3856 (4.6%)	30710 (3.1%)	11362 (3.4%)
Asian	7274 (3.0%)	2386 (2.8%)	27618 (2.8%)	9753 (2.9%)
Other	7646 (3.2%)	2607 (3.1%)	29792 (3.0%)	10649 (3.2%)
**AGE**				
<30 year	22133 (9.1%)	8847 (10.5%)	58766 (5.8 %),	25224 (7.5%)
30-50 years	57331 (23.6%)	20848 (24.8%)	204844 (20.4%)	75973 (22.6%)
50-70 years	102787 (42.2%)	34350 (40.8%)	473973 (47.12%)	155427 (46.3%)
>70 years	60651 (25.0%)	20656 (24.6%)	268254 (26.7%)	85492 (25.5%)
**GENDER**				
Male	116512 (48.0%)	38106 (45.3%)	446158 (44.4%)	148785 (44.3%)
Female	126350 (52.0%)	46026 (54.7%)	559594 (55.6%)	187043 (55.7%)
**INSURANCE**				
Commercial	122574 (50.5%)	45399 (54.0%)	543405 (54.0%)	194823 (58.0%)
Medicare/Medicaid	110615 (45.5%)	36084 (42.9%)	432643 (43.0%)	137703 (41.0%)
Others	9713 (4.0%)	3801 (4.52%)	29778 (2.96%)	11930 (3.6%)
**MODALITY**				
Radiograph	119361 (49.1%)	55510 (66.0%)	258772 (25.7%)	131184 (39.1%)
Computed Tomography	78455 (32.3%)	43599 (51.8%)	233228 (23.2%)	127551 (38.0%)
Magnetic Resonance Imaging	9647 (4.0%)	6744 (8.0%)	233831 (23.3%)	136628 (40.7%)
Ultrasound	28961 (11.9%)	18340 (21.8%)	152469 (15.2%)	90481 (26.9%)
Nuclear Medicine Study	940 (0.4%)	858 (1.0%)	33015 (3.3%)	27684 (8.2 %)
Fluoroscopy	4980 (2.1%)	3814 (4.5%)	24873 (2.5%)	18957 (5.6%)

eTable. This table demonstrates the demographics of our study population including race, age, gender, insurance coverage, and imaging modality

© 2025 Pollock JR et al. *JAMA Network Open*.
