# Supplementary material for: Inpatient and Outpatient Radiology Report Access After the 21st Century Cures Act
Source: JAMA Netw Open. 2025 Aug 27;8(8):e2528683. doi: 10.1001/jamanetworkopen.2025.28683 (PMC12391978; doi:10.1001/jamanetworkopen.2025.28683)
Supplement: Supplement 2. — Data Sharing Statement [file jamanetwopen-e2528683-s002.pdf]

Data Sharing Statement

Pollock. Inpatient and Outpatient Radiology Report Access After the 21st Century Cures Act. *JAMA Netw Open*. Published August 22, 2025. doi:10.1001/jamanetworkopen.2025.28683

Data
Data available: No
